# Motion analysis using wearable wireless sensors to support treatment of diseases in the elderly

**DOI:** 10.1186/2193-1801-4-S2-P1

**Published:** 2015-11-27

**Authors:** Calvin Hoi-kok Cheung, Timothy Kin-sang Lee

**Affiliations:** Engineering Discipline, Pro-Act (Electrical) (Kwai Chung), Hong Kong; Engineering Discipline, Pro-Act (Electronics) (Kowloon Bay), Hong Kong

## Background

In the near future, the elderly population (over the age of 65) in most developed countries is expected to increase and approach one fifth of the total population [1]. To reduce pressure on the health care system, the elderly can stay in their homes and modern wearable wireless sensor technologies can be used to monitor their health. One of the functions of our healthcare monitoring system is to detect the hand tremor that is a common symptom of Parkinson's disease. Elderly patients often have difficulty remembering the extent of their hand tremor over time and struggle to report it clearly to their doctors during consultations. Our system precisely records the condition and summarises the information to assist doctors in making their assessments.

## Methods

We attach a set of wearable wireless sensors to different parts of the body of an elderly patient. The sensors capture motion and send data to a computer for analysis. We analyse signals from five sensors attached to the chest, wrists and thighs. To monitor hand tremor, we analyse the velocity signals of the wrist sensors. If the signal energy for frequencies ranging from 3 to 8 Hz is higher than a reference level, we consider the patient has hand tremor. The reference level is an average of several velocity signals sampled from a man with no hand tremor. The amount of energy exceeding the reference level represents the severity of patients' tremor.

An elderly patient who is suspected to have Parkinson's disease wears the set of sensors in the daytime for a few days. A computer records and analyses the data from the sensors to generate a report summarising the hand tremor. This report can assist doctors in making the best treatment plan.

In addition to monitoring the hand tremor, we also analyse the orientation signals from the chest and the two thigh sensors. We note that different postures have different signatures in the signals [2, 3] and we can detect standing, sitting and lying. These posture results are saved as activities of daily living data for future analysis.

## Results

Our system manages to capture hand motion and differentiate tremor cases from normal cases. A daily report can be generated to summarise the percentage of time hand tremor is detected. This information can help doctors to make a better treatment plan for the elderly. A few tests were conducted on several individuals and the tremor conditions were successfully recorded and identified. We also plan to use this technology to analyse the relationship between different medications, medication dosages and hand tremor severity. We expect this analysis will help doctors to determine which medication and dosage is most effective for treating hand tremor. We were also able to detect standing, sitting and lying postures by analysing the sensor orientation signals. These posture results may help to identify behavioural abnormalities associated with dementia.

## Conclusions

In conclusion, we made use of a wireless sensor network to monitor hand tremor. A report was generated summarising the tremor of patients with Parkinson's disease to help doctors determine the most suitable treatment plan. We tested our method on several individuals and found it to be successful in recording and analysing tremor. Note that the test is not a clinical trial but a preliminary test only. In addition, we also detected different postures including standing, sitting and lying. These results may help doctors to identify behavioural abnormalities that may be used as reference information for early prediction or detection of some diseases of the elderly, including dementia.
